# Increasing leaf hydraulic conductance with transpiration rate minimizes the water potential drawdown from stem to leaf

**DOI:** 10.1093/jxb/eru481

**Published:** 2014-12-29

**Authors:** Kevin A. Simonin, Emily Burns, Brendan Choat, Margaret M. Barbour, Todd E. Dawson, Peter J. Franks

**Affiliations:** ^1^Department of Biology, San Francisco State University, San Francisco, CA 94132, USA; ^2^Save The Redwoods League, 111 Sutter Street, 11th Floor, San Francisco, CA 94104, USA; ^3^University of Western Sydney, Hawkesbury Institute for the Environment, Locked Bag 1797, Penrith 2751, NSW, Australia; ^4^Faculty of Agriculture and Environment, University of Sydney, NSW 2006, Australia; ^5^Department of Integrative Biology, University of California-Berkeley, Berkeley, CA 94720, USA

**Keywords:** Leaf hydraulic conductance, leaf water potential, stem water potential, stomatal conductance, transpiration, water relations.

## Abstract

Leaf hydraulic conductance (*k*_leaf_) is a central element in the regulation of leaf water balance but the properties of *k*_leaf_ remain uncertain. Here, the evidence for the following two models for *k*_leaf_ in well-hydrated plants is evaluated: (i) *k*_leaf_ is constant or (ii) *k*_leaf_ increases as transpiration rate (*E*) increases. The difference between stem and leaf water potential (ΔΨ_stem–leaf_), stomatal conductance (*g*_s_), *k*_leaf_, and *E* over a diurnal cycle for three angiosperm and gymnosperm tree species growing in a common garden, and for *Helianthus annuus* plants grown under sub-ambient, ambient, and elevated atmospheric CO_2_ concentration were evaluated. Results show that for well-watered plants *k*_leaf_ is positively dependent on *E*. Here, this property is termed the dynamic conductance, *k*_leaf(E)_, which incorporates the inherent *k*_leaf_ at zero *E*, which is distinguished as the static conductance, *k*_leaf(0)_. Growth under different CO_2_ concentrations maintained the same relationship between *k*_leaf_ and *E*, resulting in similar *k*_leaf(0)_, while operating along different regions of the curve owing to the influence of CO_2_ on *g*_s_. The positive relationship between *k*_leaf_ and *E* minimized variation in ΔΨ_stem–leaf_. This enables leaves to minimize variation in Ψ_leaf_ and maximize *g*_s_ and CO_2_ assimilation rate over the diurnal course of evaporative demand.

## Summary

Under well-watered conditions leaf hydraulic conductance increases with transpiration rate. This reduces the water potential gradient in leaves and potentially improves productivity under daily variation in evaporative demand.

## Introduction

The co-variation between leaf water potential (Ψ_leaf_), transpiration rate (*E*), stomatal conductance (*g*_s_), and CO_2_ assimilation rate (*A*) at any instant in time, or integrated over the life of a leaf, is considered to be strongly influenced by the water permeability of the cells that define the leaf hydraulic network (e.g. Sôber, 1997; Franks, 2006; Sack and Holbrook, 2006; Brodribb *et al.*, 2007; Simonin *et al.*, 2012). The cell types that comprise the leaf hydraulic network vary greatly in structure and function. At one extreme are the relatively rigid, dead, xylem cells comprising solely of cell wall, and at the other extreme are the live parenchyma cells of the extra-xylary tissue. At the whole-leaf level these two cell types in combination represent, on average, ~50% of the total liquid-phase conductance to water flow along the transpiration stream between the roots and sites of evaporation within the leaves of a plant (Nardini *et al.*, 2005*a*; Sack *et al.*, 2005). Thus, in response to variation in water availability or demand, the conductance of both the leaf xylem and extra-xylary pathways strongly influence the changes in liquid flux.

**Fig. 1.**
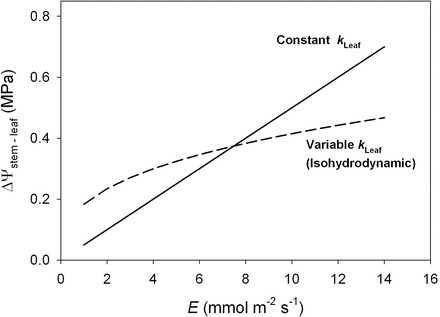
Two models describing the water potential drawdown from stem to leaf (ΔΨ_stem–leaf_, MPa) in response to changes in transpiration rate (*E*, mmol m^–2^ s^–1^). The solid black line represents a model scenario when leaf hydraulic conductance (*k*_leaf_, mmol m^–2^ s^–1^ MPa^–1^) is constant, whereas the dashed line represents a model scenario when *k*_leaf_ is positively dependent on *E*, commonly referred to as isohydrodynamic conditions.

Models used to describe changes in leaf hydraulic conductance (*k*_leaf_), in response to variation in water availability or demand, are often based on dynamics previously observed in stems by emphasizing xylem vulnerability to cavitation as Ψ_leaf_ decreases (e.g. Brodribb and Holbrook, 2006). According to this xylem-centric framework, *k*_leaf_ is at a maximum when leaves are well hydrated (i.e. high Ψ_leaf_). As *E* increases, *k*_leaf_ may stay relatively constant, with Ψ_leaf_ decreasing until a threshold is reached that results in the formation of xylem emboli, followed by a rapid decline in *k*_leaf_ with any further decrease in Ψ_leaf_ (e.g. Blackman *et al.*, 2009; Johnson *et al.*, 2009; Martorell *et al.*, 2014). Under this scenario, increases in *E* would increase the driving gradient for water flow across a leaf. In other words, above the cavitation threshold a positive linear relationship is expected between the water potential difference between stem and leaf (ΔΨ_stem–leaf_) and *E* (e.g. Fig. 1, solid black line).

Xylem-focused models have been extremely useful for characterizing the extent to which emboli induced by water stress begin to limit leaf gas exchange and primary productivity (e.g. Salleo *et al.*, 2001; Sperry *et al.*, 2002; Cochard *et al.*, 2002; Johnson *et al.*, 2012). However, it is becoming increasingly apparent that changes in how water is transported through the extra-xylary component of the transpiration stream can lead to large, sustained changes in *k*_leaf_ before or even after the onset of cavitation (e.g. Matzner and Comstock, 2001; Lo Gullo *et al.*, 2005; Nardini *et al.*, 2005*b*; Cochard *et al.*, 2007; Sellin and Kupper, 2007; Sellin *et al.*, 2008; Scoffoni *et al.*, 2008; Pou *et al.*, 2013; Shatil-Cohen *et al.*, 2011). These observations suggest an additional mechanism driving the co-variation between Ψ_leaf_, *E*, *g*_s_, and *k*_leaf_ that cannot be explained by models that emphasize xylem vulnerability to cavitation. As the vast majority of carbon gain and water use occurs when leaf water potentials are above the water potential thresholds that lead to significant loss of hydraulic conductance via xylem embolism, there is a need for a greater understanding of the mechanisms governing the co-variation between Ψ_leaf_, *E*, *g*_s_, *k*_leaf_, and ultimately *A* when leaves are well hydrated. A comprehensive quantification of the relationship between *k*_leaf_, *g*_s_, and *E* is crucial in vegetation models that incorporate hydraulic processes.

Although stomata and the leaf hydraulic network control two distinct phases of water transport across the soil–plant–atmosphere continuum (i.e. vapour and liquid flux), coordination between these two regulatory systems must exist if leaves are to maintain physiologically favourable water contents and avoid desiccation while maximizing carbon gain. If the goal is to maximize carbon gain, then leaves need to keep stomata open over a broad diurnal range of evaporative demand. The problem for any leaf with fixed *k*_leaf_, or one in which *k*_leaf_ only declines with increasing *E*, is that the water potential drawdown (ΔΨ_stem–leaf_) increases with *E* which, via the hydraulic feedback loop, tends to reduce *g*_s_ and CO_2_ assimilation rate (Cowan, 1977; Buckley, 2005; Franks *et al.*, 2007). One solution to this problem is for leaves to vary *k*_leaf_ positively with *E* to minimize the change in ΔΨ_ stem–leaf_, maintaining isohydrodynamic conditions. Leaves operating with this mechanism will show a nonlinear relationship between ΔΨ_stem–leaf_ and *E* where the ratio of *E* to ΔΨ_stem–leaf_ increases as *E* increases (Fig. 1, dashed line). In other words, a positive correlation between *k*_leaf_ and *E* would reduce daytime depressions in Ψ_leaf_ and increase the maximum potential *g*_s_ and *A* for a given leaf to air vapour pressure difference (VPD).

Isohydrodynamic behaviour has been shown for whole-plant hydraulic conductance (Franks *et al.*, 2007). Reports of relatively constant ΔΨ_stem–leaf_ (Black, 1979; Brodribb and Holbrook, 2003) suggest that the same mechanism may operate at the leaf level. Here the hypothesis that variation in *g*_s_ and *k*_leaf_ are connected through optimization of the water potential drawdown across a leaf is tested. Specifically, it is predicted that variation in *k*_leaf_ occurs to maximize leaf gas exchange while minimizing variation in ΔΨ_stem–leaf_. Using *in situ* measurements of *k*_leaf_ taken on species grown under well-watered conditions in a common garden and on plants grown under three different atmospheric CO_2_ concentrations, two possible models describing the mechanistic links between Ψ_leaf_, *E*, *g*_s_, and *k*_leaf_ in well-hydrated plants are tested: (i) constant *k*_leaf_, versus (ii) increasing *k*_leaf_ as *E* increases.

## Materials and methods

### Common garden

Four deciduous and two evergreen temperate forest tree species were evaluated; three angiosperms, *Acer macrophyllum* Pursh (Aceraceae), *Populus fremontii* Watson (Salicaceae), and *Quercus kelloggii* Newberry (Fagaceae), and three gymnosperms, *Metasequoia glyptosrtoboides* Hu and Cheng (Cupressaceae), *Pinus ponderosa* P. Laws (Pinaceae), and *Sequoia sempervirens* D. Don (Cupressaceae). Four or five saplings of each species were grown from seed in 40 l pots and transferred to a single site (common garden), in full sun, on a ridge top at the University of California Botanical Garden (32°52′N 122°14′W, ~256 m elevation) between 1–7 March 2007. Individual saplings from each species were randomized spatially throughout the common garden. Saplings ranged from ~1.5–2.5 m in height. Plants were kept well watered using drip irrigation. Data were collected when leaves were fully expanded, ~1 month after the start of leaf emergence, which occurred in late May/early June of 2008 and 2009 for *A. macrophyllum*, *M. glyptostroboides*, *P. fremontii*, and *Q. kelloggii* and mid to late July for *P. ponderosa*, and *S. sempervirens*. Air temperature and relative humidity were measured with a Li-1600 steady-state porometer (Licor Inc., Lincoln NE, USA) in close proximity to the leaves in which gas exchange and water potential were measured. Photosynthetically active radiation intercepted by the adaxial surface of the leaf was measured with a quantum sensor (Model Li-190SB, Licor Inc., Lincoln NE, USA).

Diurnal variation in leaf hydraulic conductance (*k*_leaf_; mmol m^–2^ s^–1^ MPa^–1^) for sun-exposed leaves was measured on four to five individuals of each species using the *in* situ evaporative flux method (*in situ* EFM), with *k*_leaf_ calculated as (Brodribb and Holbrook, 2003):

$$k_{\text{leaf}}=E/\Delta \Psi_{\text{stem-leaf}}$$ (1)

where *E* is the transpiration rate (mmol m^–2^ s^–1^), and ΔΨ_stem–leaf_ is the difference between stem xylem water potential (Ψ_stem_; MPa) and leaf water potential (Ψ_leaf_; MPa). This *in situ* technique required sampling two adjacent leaves, one of which was used to measure Ψ_stem_ whereas the adjacent leaf was sampled for *E* and Ψ_leaf_. Leaves used as an assay for Ψ_stem_ were covered in plastic film and aluminium foil on the evening before the measurement period to ensure equilibration between the covered Ψ_leaf_ and Ψ_stem_. Transpiration rate (*E*) was measured with the Li-1600 porometer. Owing to the open crown structure of the saplings, the wide spacing between trees, and the windy ridge top exposure of the common garden, it was assumed that leaf boundary layer conductance (*g*_b_) was much greater than *g*_s_. Additionally, during each measurement of *E*, leaf orientation, ambient humidity, and radiation interception was conserved. Therefore, *E* measured by the Li-1600 was likely to be similar to the actual *E* immediately before the measurement. While measuring *E*, the water potential of the adjacent covered leaf was sampled as a proxy for Ψ_stem_. Immediately following determination of *E,* the uncovered leaf was excised, wrapped in plastic, and placed in a Scholander-type pressure chamber for determination of Ψ_leaf_ (Soil Moisture Equipment Corp., Santa Barbara CA, USA). Balancing pressure was recorded when xylem sap reached the cut stem surface, as verified by a dissecting scope at ×25 magnification. *E*, Ψ_stem_, and Ψ_leaf_ were measured every ~2.5h over the course of a 14–18h period, beginning at pre-dawn (0400–0500h). Regression analysis was used to evaluate the co-variation between ΔΨ_stem–leaf_, *E*, *g*_s_, and *k*_leaf_ over a diurnal cycle of evaporative demand. Regression analyses were performed using SigmaPlot (Version 11; Systat Software Inc., San Jose, CA, USA).

### Growth chamber experiment

ΔΨ_stem–leaf_, *E*, *g*_s_, *A*, and *k*_leaf_ were measured at two light levels (509±11.5 and 1310±26.4 µmol m^–2^ s^–1^) for *Helianthus annuus* plants grown under sub-ambient (194±35 ppm), ambient (450±46 ppm), and elevated CO_2_ (1027±74 ppm). *H. annuus* plants were grown in growth chambers located in the Controlled Environment Facility at the Center for Carbon, Water, and Food at the University of Sydney. Ten plants were grown under each CO_2_ concentration at 900±50 µmol m^–2^ s^–1^ of photosynthetically active radiation (PAR). Ambient CO_2_ concentrations were monitored using an isotope ratio infrared spectrometer (G1101-i, Picarro, CA, USA) that cycled through each room every 10min. Using the method outlined above, *g*_s_ and *E* were measured with a portable photosynthesis system fitted with a large leaf cuvette that enclosed the entire leaf (Walz-USA, Pepperell MA, USA). Water potential was measured using a Scholander-style pressure chamber (Soil Moisture Equipment Co., Santa Barbara CA, USA) and *k*_leaf_ was calculated from Eqn 1. Measurements were taken after the leaves were in the cuvette for ~40min during stable *g*_s_, *E*, *A*, and *T*_leaf_. After the 40min period leaves were cut from the stem, removed from the cuvette, and covered in plastic for water potential measurements with the Scholander-style pressure chamber as described above. Projected leaf area was measured digitally using the software program Image J (US National Institutes of Health, Bethesda, Md). Two leaves were used as an assay for Ψ_stem_ using the method outlined above. Leaves directly above and below the leaf in the cuvette were selected and the average of the two water potential measurements was taken as Ψ_stem_. In all cases the distal leaf showed a slightly more negative Ψ_stem_, ~0.02–0.04MPa. Measurements of Ψ_leaf_, *E*, *g*_s_, *A*, and *k*_leaf_ were made at 450 ppm CO_2_ inside the cuvette at two light levels, ~500 and 1300 µmol m^–2^ s^–1^ PAR, and a relatively constant leaf temperature of 24.7±0.2 °C by varying air temperature and ambient humidity inside the cuvette. In total, five leaves from each CO_2_ growth environment were measured at each light level (~500 and 1300 µmol m^–2^ s^–1^ PAR), all at 450 ppm CO_2_. All statistical analyses were performed using SigmaPlot and JMP (v.4.0.4; SAS Institute, Cary, NC, USA). ANCOVA was used to test for main and interactive effects of growth CO_2_ (low, medium, and high), light (low, high), and VPD on g_s_ and *A*. An ANCOVA was also used to test for main and interactive effects of CO_2_ (low, medium and high), light (low, high), and *E* (covariate) on *k*_leaf_. Because measurements within the light treatment were done on the same plant, plants nested within the light treatment were used as a random factor (Quinn and Keough, 2002). Assumptions of normality were met.

### Estimation of *k*_leaf_ when *E*=0

As shown by Eqn 1, using the *in situ* EFM technique to measure *k*_leaf_ requires an evaporative flux and a water potential drawdown which in turn prevents direct calculation of *k*_leaf_ when *E*=0. However, theory predicts that when *E*=0, *k*_leaf_ will have some finite value. By measuring *k*_leaf_ across a broad range of *E* a linear model can be used to extrapolate to *k*_leaf_ at zero *E* (i.e. the static conductance, *k*_leaf(0)_). Linear regression was used to estimate *k*_leaf(0)_ for both gymnosperm and angiosperm trees from the common garden and *H. annuus* plants from the growth chamber experiment.

## Results

### Common garden

**Fig. 2.**
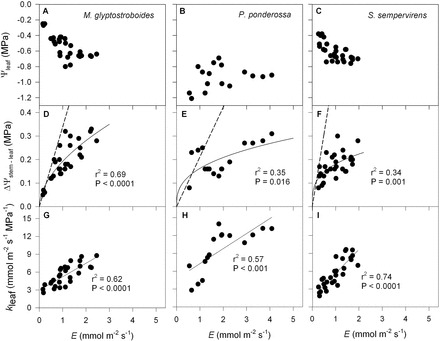
Variation in leaf water potential (Ψ_leaf_, MPa), the difference between stem and leaf water potential (ΔΨ_stem–leaf_, MPa), and leaf hydraulic conductance (*k*_leaf_, mmol m^–2^ s^–1^ MPa^–1^) as a function of transpiration rate (*E*, mmol m^–2^ s^–1^) for the three gymnosperm species from the common garden: *M. glyptostroboides* (A, D, G); *P. ponderosa* (B, E, H); and *S. sempervirens* (C, F, I). The solid black line in each panel represent the best-fit model describing the coordination between Ψ_stem–leaf_ and *k*_leaf_ with variation in *E*. The dashed lines in panels D, E, and F represent the predicted changes in ΔΨ_stem–leaf_ for a leaf that possesses the average static leaf hydraulic conductance (i.e. *k*_leaf(0)_) for each individual species._._ The coefficient of determination (r^2^) and significance (*P*) in each panel refer to the solid lines.

**Fig. 3.**
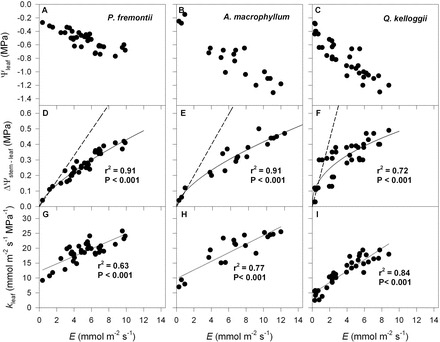
Variation in leaf water potential (Ψ_leaf_, MPa), the difference between stem and leaf water potential (ΔΨ_stem–leaf_, MPa), and leaf hydraulic conductance (*k*_leaf_, mmol m^–2^ s^–1^ MPa^–1^) as a function of transpiration rate (*E*, mmol m^–2^ s^–1^) for the three angiosperm species from the common garden: *P. fremontii* (A, D, G); *A. macrophyllum* (B, E, H); and *Q. kelloggii* (C, F, I). Solid and dashed lines, as well as r^2^ and *P* values are as for Fig. 2.

**Fig. 4.**
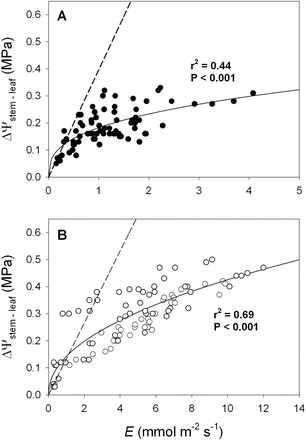
The difference between stem and leaf water potential (ΔΨ_stem–leaf_, MPa) as a function of transpiration rate (*E*, mmol m^–2^ s^–1^) for: (A) gymnosperm and (B) angiosperm tree species from the common garden. Solid lines, as well as *r* ^2^ and *P* values are as for Fig. 2. The dashed lines represent the predicted changes in ΔΨ_stem–leaf_ for a leaf that possesses the average static leaf hydraulic conductance (i.e. *k*_leaf(0)_) for the (A) gymnosperm and (B) angiosperm species.

**Fig. 5.**
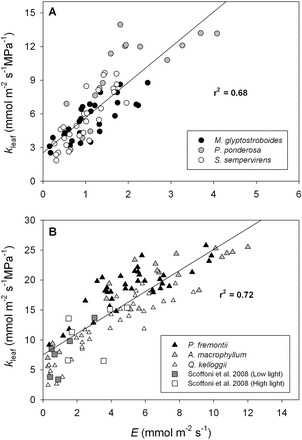
Leaf hydraulic conductance (*k*_leaf_, mmol m^–2^ s^–1^ MPa^–1^) as a function of transpiration rate (*E*, mmol m^–2^ s^–1^) for: (A) gymnosperm and (B) angiosperm tree species from the common garden. Previously published data taken from Table 2 in Scoffoni *et al.* (2008) is also shown as filled and open squares in B. Filled squares represent the *k*_leaf_ values taken when leaves were exposed to low light (<10 µmol m^–2^ s^–1^), whereas the open squares represent the *k*_leaf_ values when leaves were exposed to high light (>1000 µmol m^–2^ s^–1^; See Scoffoni *et al.* (2008) for further details). The solid line in each panel was fitted by linear regression through all the data, excluding the data by Scoffoni *et al*. (2008).

**Fig. 6.**
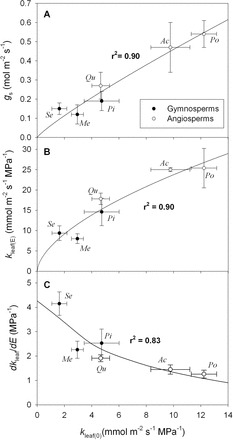
The influence of static leaf hydraulic conductance (*k*_leaf(0)_, mmol m^–2^ s^–1^ MPa^–1^) on (A) maximum daytime stomatal conductance (*g*_s_, mol m^–2^ s^–1^); (B) maximum daytime leaf hydraulic conductance (*k*_leaf(E)_, mmol m^–2^ s^–1^ MPa^–1^), and (C) the slope of the linear relationship between *k*_leaf(E)_ and *E* (i.e. the hydraulic gain) for three gymnosperm and three angiosperm species. *Se*, *Me, Pi, Qu, Ac* and *Po* are *Sequoia sempervirens, Metasequoia glyptostroboides, Pinus ponderosa, Quercus kelloggii, Acer macrophyllum,* and *Populus fremontii*, respectively. Solid lines represent the best-fit model describing the coordination between *g*_s_, *k*_leaf(E)_ and *dk*_leaf_/*dE* with variation in *k*_leaf (0)_.

A strong non-linear relationship was observed between ΔΨ_stem–leaf_ and *E* for both the gymnosperm (Fig. 2) and angiosperm species (Fig. 3) from the common garden. Although the maximum transpiration rates for the gymnosperm species were lower than the angiosperm species (Table 1), on average, the co-variation between ΔΨ_stem–leaf_ and *E* was similar between species (gymnosperms, *y*=0.18*x* ^0.35^, r^2^=0.44, *P*<0.001; angiosperms, *y*=0.14*x* ^0.48^, r^2^=0.69, *P*<0.001), with angiosperms attaining higher overall ΔΨ_stem–leaf_ and *E* (Fig. 4). Assuming liquid fluxes into the leaf and vapour phase fluxes from the leaf were in steady-state, the observed correlation between Ψ_stem–leaf_ and *E* was the result of a strong coupling between *k*_leaf_ and *E* (Table 1). A significant positive relationship was observed between *k*_leaf_ and *E* across the gymnosperm and angiosperm species from the common garden (Figs 2, 3, 5). For each species the *y*-intercept of the linear model describing the co-variation between *k*_leaf_ and *E* was greater than 0 (Figs 2, 3; Table 1). This static leaf hydraulic conductance when *E*=0 (*k*_leaf(0)_), varied greatly between angiosperm and gymnosperm species (Figs 2, 3; Table 1), with angiosperms having a higher *k*_leaf(0)_ (Fig. 5; Table 1). For the plants growing in the common garden, a higher *k*_leaf(0)_ was associated with greater maximum daytime *g*_s_, *k*_leaf_, and lower *dk*_leaf_/*dE* (Fig. 6; Table 1). The angiosperm species from the common garden operated at a higher day time maximum *g*_s_ and ΔΨ_stem–leaf_ than the gymnosperm species (Figs 4, 6; Table 1).

**Table 1.** Daytime maximum stomatal conducatance (g_s_) and transpiration (*E*)±1 standard deviation for study species from the common garden The *r* ^2^ for the linear model describing the co-variation between *k*_leaf_ and *E* including the y-intercept (*k*_leaf(0)_) and gain (*dk*_leaf_/*dE*); for every species (*P*<0.001, for all species).

	Species	*g*_s,max_ (mol m^–2^ s^–1^)	*E*_max_ (mmol m^–2^ s^–1^)	*k*_leaf(0)_ (mmol m^–2^ s^–1^ MPa^–1^)	*k*_leaf(E)_ (mmol m^–2^ s^–1^ MPa^–1^)	*dk*_leaf_/*dE* (MPa^–1^)	*k*_leaf_ vs *E*
**Angiosperms**	*Acer macrophyllum*	0.47±0.13	11.2±1.55	9.77±1.43	25±0.48	1.45±0.19	r^2^=0.77
	*Populus fremontii*	0.54±0.07	9.38±2.75	12.23±0.93	25.39±4.85	1.26±0.16	r^2^=0.63
	*Quercus kelloggii*	0.27±0.07	5.18±1.15	4.66±0.65	17.83±1.41	1.91±0.14	r^2^=0.84
**Gymnosperms**	*Metasequoia glyptostroboides*	0.12±0.05	1.93±0.42	2.95±0.44	8.01±1.20	2.26±0.35	r^2^=0.62
	*Pinus ponderosa*	0.19±0.05	2.79±0.44	4.73±1.28	14.61±3.38	2.53±0.58	r^2^=0.57
	*Sequoia sempervirens*	0.15±0.03	1.53±0.47	1.63±0.55	9.38±1.79	4.15±0.48	r^2^=0.74

Qualitatively similar diurnal trends in *g*_s_, *E*, Ψ_leaf_, Ψ_stem_, and *k*_leaf_ were observed for the angiosperm and gymnosperm species grown in the common garden. In general, from dawn to midday, Ψ_leaf_ and Ψ_stem_ decreased while *g*_s_, *E*, and *k*_leaf_ increased (Supplementary Figs S1 and S2). After midday, Ψ_leaf_ and Ψ_stem_ increased slightly, whereas *g*_s_, *E*, and *k*_leaf_ decreased (Supplementary Figs S1 and S2). Overall, plants in the common garden were well hydrated, with Ψ_stem_ and Ψ_leaf_ greater than –0.8 and –1.2MPa, respectively. The diurnal changes in Ψ_leaf_ and Ψ_stem_ followed a similar pattern such that ΔΨ_stem-leaf_ changed very little over the course of a day (Supplementary Figs S1 and S2). On average the angiosperm species showed greater diurnal variation in *g*_s_, *E*, and *k*_leaf_ compared with the gymnosperm species (Supplementary Figs S1 and S2).

### Growth chamber

**Fig. 7.**
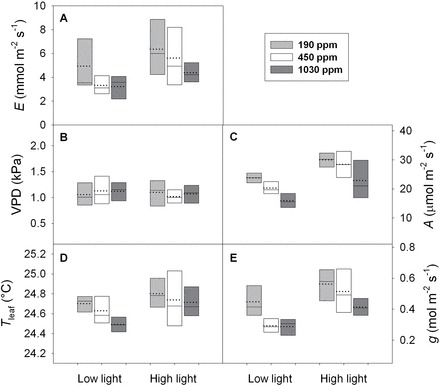
The range (indicted by boxes), median (horizontal line within boxes), and mean (dotted line within boxes) of: (A) transpiration rate (*E*, mmol m^–2^ s^–1^), (B) leaf-to-air vapour pressure difference (VPD, kPa), (C) CO_2_ assimilation rate (*A*, µmol m^–2^ s^–1^), (D) leaf temperature (*T*_leaf_, °C), and (E) leaf surface conductance, comprising the sum of stomatal and boundary layer conductances *g*_s_ and *g*_b_ (*g*, mol m^–2^ s^–1^) during measurements of leaf hydraulic conductance (*k*_leaf_) at both low (500 µmol m^–2^ s^–1^ PAR) and high light (1300 µmol m^–2^ s^–1^ PAR), for *H. annus* plants grown under ~190 (grey boxplot), 450 (open boxplot), and 1030 ppm CO_2_ (dark grey boxplot). Note that here, *g*_b_ is considered sufficiently high such that *g* ≈*g*_s_.

Across all three CO_2_ treatments a similar range of *E*, VPD, *A*, *T*_leaf_, and *g*_s_, occurred at each light level (Fig. 7A–E). Overall, PAR had the greatest significant effect on *g*_s_ (F=31.85, *P*<0.001) followed by VPD (F=8.55, *P*=0.012) and CO_2_ (F=7.7281, *P*=0.0129) with no significant interaction between PAR, CO_2_, and VPD. Across CO_2_ treatments, increases in PAR were associated with greater *g*_s_ (t=5.64, *P*<0.001; Fig. 7E). On average, plants grown under sub-ambient CO_2_ operated at higher *g*_s_ than plants from elevated CO_2_ (t=3.95, *P*=0.002) but were not statistically different from plants grown under ambient CO_2_ (t=1.89, *P*=0.08). Although VPD showed a significant main effect on *g*_s_, correlations between *g*_s_ and VPD within each CO_2_ treatment were not significantly different, which was probably due to the small range of VPD and limited number of measurements at different VPD (*n*=5) at a given light level. Both PAR (F=31.49, *P*<0.001) and CO_2_ (F=9.76, *P*<0.002) significantly influenced variation in *A*. Across treatments, increasing PAR had a positive effect on *A* (t=5.612, *P*<0.001). Similar to *g*_s_, when measured at the same atmospheric CO_2_ concentration (450 ppm), plants from the sub-ambient CO_2_ treatment showed higher *A* than plants from the elevated CO_2_ treatment (t=4.24, *P*=0.001) but were not statistically different from the ambient CO_2_ treatment (t=0.89, *P*=0.39). Using an ANCOVA to test for main and interactive effects of CO_2_, light, and *E* on *k*_leaf_, it was found that *E* had the only significant effect on *k*_leaf_ (F=16.1945, *P*=0.0027).

**Fig. 8.**
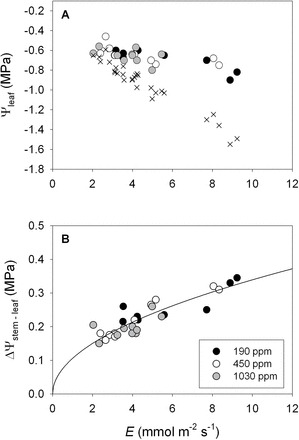
The influence of variation in transpiration rate (*E*, mmol m^–2^ s^–1^) on (A) leaf water potential and (B) the difference between stem and leaf water potential (ΔΨ_stem–leaf_, MPa) for: *H. annus* plants grown under ~190 (filled circles), 450 (open circles), and 1030 ppm CO_2_ (grey circles). The crosses in A represent the predicted leaf water potentials for a leaf that possesses the average static leaf hydraulic conductance (i.e. *k*_leaf(0)_=9.08 mmol m^–2^ s^–1^ MPa^–1^) across all three CO_2_ treatments. The solid black line in panel (B) represents the best-fit model describing the coordination between ΔΨ_stem–leaf_ and *E*.

**Fig. 9.**
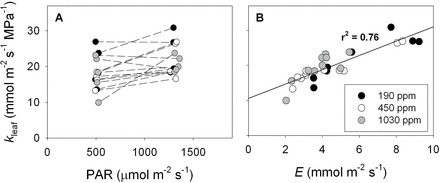
The influence of variation in: (A) photosynthetically active radiation (PAR, µmol m^–2^ s^–1^) and (B) transpiration rate (*E*, mmol m^–2^ s^–1^) on leaf hydraulic conductance (*k*_leaf_, mmol m^–2^ s^–1^ MPa^–1^). The dotted lines in A show the ‘light–*g*-effect’ on *k*_leaf_ for leaves at a common leaf to air vapour pressure difference (VPD). In all cases where greater light interception resulted in an increase in *k*_leaf_, at a common VPD, there was a light induced increase in *g* and by extension *E*.

Across all CO_2_ treatments an increase in *E* was associated with a decrease in Ψ_leaf_, although the decreases in Ψ_leaf_ were relatively minor despite relatively large variation in *E* (Fig. 8A). Similar to plants from the common garden, a strong non-linear relationship between ΔΨ_stem–leaf_ and *E* was observed across all three growth CO_2_ treatments (y=0.106×*E* ^0.507^, *r* ^2^=0.77, *P*<0.001; Fig. 8B; assuming that the when *E*=0, ΔΨ_stem–leaf_=0). This was the result of a significant positive relationship between *k*_leaf_ and *E* across all three CO_2_ treatments (y=9.70 + 2.36×*E, r* ^*2*^=0.72; Fig. 9B). The lack of interactive effects between *E* and CO_2_ on *k*_leaf_ suggests that *k*_leaf(0)_ was not significantly influenced by growth CO_2_ (Table 2). Similarly, *k*_leaf_ per unit *g*_s_ and *A* were relatively unaffected by PAR, but were significantly, positively correlated with VPD (*k*_leaf_/*g* vs. VPD: y=0.0281×*e* ^(0.5355×VPD)^, *r* ^2^=0.23, *P*=0.009; *k*_leaf_/*A* vs. VPD: y=0.4837×*e* ^(0.4634×VPD)^, *r* ^2^=0.30, *P*=0.002).

**Table 2.** Mean stomatal conducatance (*g*_s_) and transpiration (E)±1 standard deviation for H. annus plants grown under ~190, 450, and 1030 ppm CO_2_. Also shown are the hydraulic gain (dk_leaf_/dE) and the y-intercept (k_leaf_(0)) and r2 for the linear model describing the co-variation between k_leaf_ and E (k_leaf_ vs E)*P < 0.001; **P < 0.01.

Growth CO_2_ (ppm)	Mean *g*_s_ (mol m^–2^ s^–1^)	*Mean E* (mmol m^–2^ s^–1^)	*k*_leaf(0)_ (mmol m^–2^ s^–1^ MPa^–1^)	*dk*_leaf_/*d*E (MPa^–1^)	*k*_leaf_ vs *E*
190±35	0.50±0.12	5.57±2.41	9.75±2.44	2.12±0.41	r^2^=0.80*
450±46	0.40±0.15	0.54±0.07	9.38±2.75	12.23±0.93	r^2^=0.91*
1030±74	0.35±0.09	0.27±0.07	5.18±1.15	4.66±0.65	r^2^=0.70**

## Discussion

**Fig. 10.**
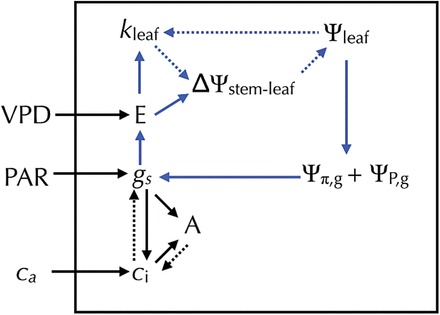
Model diagram showing the coordination between transpiration rate (*E*), the difference between stem and leaf water potential (ΔΨ_stem–leaf_), leaf hydraulic conductance (*k*_leaf_), stomatal conductance (*g*_s_, where boundary layer conductance *g*_b_ is sufficiently high for *g*_s_ to dominate), and CO_2_ assimilation rate (*A*). The blue lines represent the hydraulic feedback loop between *g*_s_ and *E.* Solid lines represent a positive relationship between parameters and dotted lines represent a negative relationship. The positive relationship between *E* and *k*_leaf_ is the predicted relationship based on data gathered from *H. annuus* plants grown under low, medium, and high CO_2_ concentrations, and three gymnosperm and angiosperm tree species growing in a common garden (see Methods for more detail). The black box indicates the boundary between leaf processes and external environmental variables. (VPD, leaf-to-air vapour pressure difference; PAR, photosynthetically active radiation; *C*_a_, atmospheric CO_2_ concentration, *C*_i_, leaf internal CO_2_ concentration, Ψ_π,g_, is the guard cell osmotic pressure, and Ψ_P,g_, guard cell turgor pressure).

The data presented here do not support a constant *k*_leaf_ model, under well-watered conditions, and instead are consistent with a hydraulic mechanism whereby *k*_leaf_ increases with *E*. Additionally, here it is shown that a positive dependence of *k*_leaf_ on *E* results in a dynamic coupling between *k*_leaf_ and *g*_s_ that ultimately minimizes the water potential drawdown across the leaf (i.e. ΔΨ_stem–leaf_) which, via the hydraulic feedback loop, increases the maximum potential *g*_s_ and *A* for a given VPD (Fig. 10).

### Static vs dynamic leaf hydraulic conductance

At steady-state, the coupling between liquid and vapour-phase water flux can be described as:

$$k_{\text{leaf}}(\Delta \Psi_{\text{stem-leaf}})=g(w_{\text{i}}-w_{\text{a}})=g(e_{\text{i}}-e_{\text{a}})/P$$ (2)

where *g* is the sum of stomatal (*g*_s_) and boundary layer conductance (*g*_b_) to water vapour in series, *w*_i_ and *w*_a_ are the mole fractions of water vapour (mol mol^–1^) inside the leaf and of the ambient atmosphere, *e*_i_ and *e*_a_ are the vapour pressures of water inside the leaf and in ambient air, and *P* is atmospheric pressure. The term (*e*_i_–*e*_a_)/*P* is commonly referred to as ‘VPD’. According to this steady-state description, when *g*_s_<<*g*_b_, up- or down-regulation of *E* via feedback responses of stomata to VPD can occur via changes in ΔΨ_stem–leaf_, *k*_leaf_, or both.

If *k*_leaf_ is static or only decreases as *E* increases then changes in *g*_s_ and *E*, before water-stressed-induced xylem embolism, would require large changes in ΔΨ_stem–leaf_ and ultimately a positive linear correlation between ΔΨ_stem–leaf_ and *g*(*w*_i_ – *w*_a_) or *E* (Fig. 1). From Eqn 2, when *k*_leaf_ is static, the ratio *g*(*w*_i_–*w*_a_)/ΔΨ_leaf_ remains constant. Eventually, increases in *g*(*w*_i_–*w*_a_) may lower leaf water potential sufficiently to induce cavitation and embolisms, reducing *k*_leaf_ and resulting in a positive feedback on ΔΨ_stem–leaf_ (Sperry, 2000). In this scenario *g*(*w*_i_–*w*_a_)/ΔΨ_leaf_ is negatively correlated with *E*. Because increasing ΔΨ_stem–leaf_ reduces maximum potential *g* and by extension CO_2_ assimilation rate (Buckley, 2005; Franks *et al.*, 2007), a control system that relies solely on a constant or decreasing *k*_leaf_ constrains carbon gain to occur within a relatively narrow range of low evaporative demand and high water availability. One way to avoid large drops in Ψ_leaf_ and *g* over a broad range of evaporative demand is to vary *k*_leaf_ positively with *E*. This dynamic coupling between *E* and *k*_leaf_ is represented here by the term *k*_leaf(E)_, which is the leaf hydraulic conductance for a given magnitude of *E*. Here, *k*_leaf(E)_ represents the dynamic hydraulic conductance.

Under relatively well-watered conditions no support was found for the hypothesis that *g*(*w*_i_–*w*_a_)/ΔΨ_leaf_ is constant or decreases with *E*. In fact the opposite relationship was observed: as *E* increased *g*(*w*_i_–*w*_a_)/ΔΨ_leaf_ increased (e.g. Figs 2, 3 and 8). As shown by Eqn 2, a positive dependence of *k*_leaf_ on *E* can lead to increasing *g*(*w*_i_–*w*_a_)/ΔΨ_leaf_ as *E* increases. Therefore, these results provide strong evidence that the relationship between *k*_leaf_, *g*_s_, and CO_2_ assimilation rate, in response to short-term changes in evaporative demand (VPD), is the result of a positive dependence of *k*_leaf_ on *E* (Fig. 10).

### Co-variation between ΔΨ_stem–leaf_, *E*, *g*_s_, and *k*_leaf_ over a diurnal cycle of evaporative demand

Previous research has provided evidence that diurnal variation in *k*_leaf_ can be partially attributed to circadian regulation (Nardini *et al.*, 2005*b*; Lo Gullo *et al.*, 2005). Here evidence is provided that a positive correlation between *k*_leaf_ and *E* is another factor influencing the coupling between *k*_leaf_ and *g*_s_ over a diurnal cycle of evaporative demand. Despite relatively large diurnal variation in *g*_s_ and *E* for the gymnosperm and angiosperm species growing in a common garden, only minor variation was observed in ΔΨ_stem–leaf_ across a large range in *E.* Until now, isohydrodynamic behaviour, or a relatively constant water potential gradient, has only been explained by a mechanism occurring at the whole plant level, from root to leaf, over seasonal changes in soil water availability (Franks *et al.*, 2007). The data presented here suggest that, under well-watered conditions, isohydrodynamic behaviour is common at the leaf level (e.g. Figs 2, 3, 8b). As described by Eqn 1 and 2, a minor variation in ΔΨ_stem–leaf_ over a large diurnal range in *E* and *g*_s,_ can occur if *k*_leaf_ is positively dependent on *E.*

Similar to an electrical circuit that maintains an electrical conductance even when there is no current, if a hydraulic connection exists between plants and the atmosphere then leaves will maintain the capacity to transport water, even when *E*=0. In other words, whether *k*_leaf_ is dynamically coupled to *E* (*k*_leaf(E)_) or static, it has a finite value when *E*=0, i.e. *k*_leaf(0)_ (see Methods). Here it is shown that, for the well-watered gymnosperm and angiosperm tree species in the common garden, this inherent capacity to transport water is greater for the angiosperm species than the gymnosperms (Fig. 6), and positively correlated with daytime maximum stomatal conductance (Fig. 6A). These patterns are consistent with the well-documented ‘coordination’ of hydraulic and gas exchange capacity across species (e.g. Meinzer and Grantz, 1990; Meinzer and Grantz, 1991; Sperry and Pockman, 1993; Winkel and Rambal, 1993; Meinzer *et al.*, 1995; Andrade *et al.*, 1998; Maherali *et al.*, 1997; Mencuccini and Comstock, 1999; Mencuccini, 2003; Brodribb *et al.*, 2005).

### Influence of atmospheric CO_2_ on the co-variation between ΔΨ_stem–leaf_, *E*, *g*_s_, *A*, and *k*_leaf_

Across all three growth CO_2_ treatments a strong non-linear relationship was observed between ΔΨ_stem–leaf_ and *E* where *g*(*w*_i_–*w*_a_)/ΔΨ_leaf_ increased as *E* increased. As with plants from the common garden experiment, this trend can be attributed to a positive correlation between *k*_leaf_ and *E* which was relatively decoupled from variation in PAR (Fig. 9). Recent research relying on the evaporative flux method provides further evidence that *k*_leaf_ can be up-regulated as transpiration increases (e.g. Scoffoni *et al.*, 2008; Guyot *et al.*, 2011). In this previous work, unlike the present evaluation, increases in transpiration rate were driven by a *g*_s_ light response. As reported here, a positive dependence of *k*_leaf_ on *E* can occur independent of variation in light availability. This suggests that an alternative mechanism is necessary to describe the coordination between *k*_leaf_ and light. For example, an isohydroynamic model predicts greater *k*_leaf_ as light interception increases if increased energy absorption results in greater *E* (see Figs 9 and 10).

The positive linear model describing the relationship between *k*_leaf_ and *E* was similar across the CO_2_ treatments such that *k*_leaf(0)_ was relatively conserved between CO_2_ treatments (Fig. 9B, Table 2). However, variation in growth CO_2_ influenced where plants operated along the linear model describing the co-variation between *k*_leaf_ and *E*, with a lower maximum *k*_leaf_ and *E* at high ambient CO_2_. Corresponding with this, average *g*_s_ was lower in the elevated CO_2_ treatment relative to the sub-ambient CO_2_ treatment, consistent with many studies on plants growing under different atmospheric CO_2_ concentrations (e.g. Morison and Gifford, 1983; Cure and Acock, 1986; Tolley and Strain, 1985; Morison and Lawlor, 1999).

The relative stability of *k*_leaf(0)_ across sub-ambient, ambient, and elevated CO_2_ treatments despite a significant decrease in *g*_s_ between sub-ambient and elevated CO_2_ is consistent with previous research. Across species, the sensitivity of maximum stomatal conductance (*g*_s(max)_) to variation in atmospheric CO_2_ (*c*_a_) seems to be strongly non-linear whereby the sensitivity of *g*_s(max)_ to changes in *c*_a_ increases at low *c*_a_ (Beerling and Woodward, 1997; Franks and Beerling, 2009; Franks *et al.*, 2012). Recent research also suggests that the relative differences in *g*_s_ between plants grown under sub-ambient, ambient, and elevated CO_2_ is less for plant species that possess an inherently high *g*_s_ (Franks *et al.*, 2012). Additionally, across species, there is a strong non-linear relationship between *g*_s_ and *k*_leaf_, when measured at a common VPD, whereby *dg*_s_/*dk*_leaf_ increases as *g*_s_ increases (Franks, 2006). Taken together, this previous research suggests that plants with an inherently high *g*_s_ and *k*_leaf_ will show relatively minor adjustments in *g*_s(max)_ and *k*_leaf(0)_ when exposed to elevated CO_2_, as shown here with the *H. annuus* plants. Similarly, recent research on soybean suggests that *k*_leaf_ is relatively insensitive to elevated CO_2_ (700 ppm) despite decreases in *g*_s_ at elevated CO_2_ (Locke et al., 2013). Further research, including more species and greater ranges of CO_2_, is necessary to better understand the influence of elevated atmospheric CO_2_ on the coordination between *E*, *k*_leaf_, g_s_, and *A*.

### The hydraulic gain, *dk*_leaf_/*dE*, and the sensitivity of *g*_s_ to *E*

Previous research has clearly demonstrated a hydromechanical basis for stomatal movement whereby changes in the maximum potential aperture of stomata and by extension *g*_s_ are strongly influenced by changes in bulk leaf water status i.e. Ψ_leaf_ (see reviews by Franks, 2004; Buckley, 2005). This hydraulic coupling between maximum potential *g*_s_ and Ψ_leaf_ results in a hydromechanical control system that is strongly influenced by both *E* and *k*_leaf_. For example, a hydromechanical stomatal control system that includes a positive dependence of *k*_leaf_ on *E* will reduce the sensitivity of Ψ_leaf_ to variation in *E*, when compared with a constant *k*_leaf_ (e.g. Fig. 8A) This can be shown mathematically by: $\Psi_{\text{leaf}}=\Psi_{\text{stem}}-\left(\frac{E}{k_{\text{leaf(0)}}\text{+}\left(\frac{dk_{\text{leaf}}}{dE}\times E\right)}\right)$ for a dynamic *k*_leaf_ model compared with $\Psi_{\text{leaf}}=\Psi_{\text{stem}}-\left(\frac{E}{k_{\text{leaf(0)}}}\right)$ for a static *k*_leaf_ model. This damping of variation in Ψ_leaf_ as *E* changes is expected to reduce the sensitivity of *g*_s_ to changes in VPD via the hydraulic feedback loop (Fig. 10). In other words, the rate and direction of change in the ratio of *k*_leaf_ on *E* (here this is termed the hydraulic gain; *dk*_leaf_/*dE*) is a good index of the sensitivity of leaf water status (i.e. Ψ_leaf_) and *g*_s_ to a change in *E*.

**Fig. 11.**
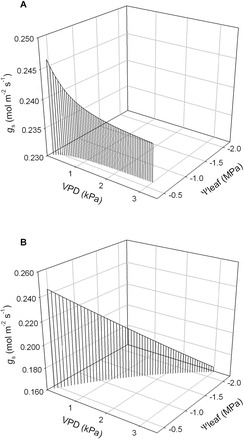
Model simulations comparing the relationship between leaf water potential (Ψ_leaf_), leaf to air vapour pressure difference (VPD), and stomatal conductance (*g*_s_) when: (A) leaf hydraulic conductance (*k*_leaf_) is positively dependent on transpiration rate (*E*), based on the empirical relationship observed across the gymnosperm species in the common garden experiment (see Fig. 5) where *k*_leaf_=2.5+3.2×*E*, and (B) a constant *k*_leaf_ based on the y-intercept of the combined gymnosperm data (i.e. *k*_leaf(0)=_2.5). Note that in A, Ψ_leaf_ remains high as VPD increases, whereas in (B) Ψ_leaf_ declines substantially with increasing VPD.

Using a hydromechanical model of *g*_s_, originally developed by Franks and Farquhar (1999) and further modified by Franks *et al.* (2007) to accommodate isohydrodynamic behaviour (i.e. decrease in ΔΨ_stem–leaf_/*E*, as *E* increases), the impact of a dynamic conductance (*k*_leaf(E)_), as compared with a static *k*_leaf_, on the sensitivity of *g*_s_ to changes in VPD was evaluated. The model output suggests that, for well-hydrated plants with a fixed Ψ_stem_, a positive dependence of *k*_leaf_ on *E* reduces the sensitivity of *g*_s_ to variation in VPD when compared with a constant *k*_leaf_ (Fig. 11). Previous research has provided strong empirical evidence that stomatal sensitivity to VPD is positively correlated with the daytime operating *g*_s_ under well watered conditions at low VPD, i.e.<1kPa (e.g. Oren *et al.*, 1999). Similarly, across species from the common garden a strong negative correlation was observed between *dk*_leaf_/*dE* and *k*_leaf(0)_, with *k*_leaf(0)_ positively correlated with daytime maximum *g*_s_ (Fig. 6A, C). Taken together, the steady-state stomatal feedback control model proposed by Franks *et al.* (2007) and the negative correlation between *dk*_leaf_/*dE* and *k*_leaf(0)_ observed here provide a mechanistic framework for evaluating empirical correlations between stomatal sensitivity to VPD and daytime operating *g*_s_ under well-watered conditions at low VPD. Further research is needed to better characterize the coordination between *k*_leaf(0)_, *dk*_leaf_/*dE*, maximum *g*_s_, and stomatal sensitivity to VPD across plants spanning a wide range in maximum potential *g*_s_.

### Possible processes underlying the variable *k*_leaf_ mechanism

It is now well recognized that *k*_leaf_ and leaf gas exchange are strongly influenced by variation in leaf vein traits (e.g. Brodribb *et al.*, 2007; Blonder *et al.*, 2011; Sack and Scoffoni, 2013). Although changes in xylem structure will directly impact the inherent hydraulic capacity of a leaf (e.g. *k*_leaf(0)_) it is unlikely that short-term increases in *E* and *k*_leaf,_ when leaves are well-hydrated, are driven by up-regulation of xylem hydraulic conductance alone. Changes in the ion concentrations of stem xylem sap have been shown to significantly influence stem hydraulic conductance (e.g. Zwieniecki *et al.*, 2001; Nardini *et al.*, 2011). Yet, to date, this ion effect has not been found in leaves (Sack *et al.*, 2004). Instead, leaf xylem hydraulic conductance, prior to any form of xylem dysfunction, is relatively constant and independent of short-term variation in evaporative demand, excluding any temperature effects on viscosity.

In contrast to that of the leaf xylem, there is growing evidence that the hydraulic conductance of the extra-xylem pathway can ramp up or down over relatively short time scales in response to changes in a particular environmental cue, such as light, temperature or water availability (e.g. Kikuta *et al.*, 1997; Sôber, 1997; Matzner and Comstock, 2001; Lo Gullo *et al.*, 2005; Sellin and Kupper, 2005*a*,*b*; Cochard *et al.*, 2007; Sellin and Kupper, 2007; Scoffoni *et al.*, 2008; Sellin *et al.*, 2008; Johnson *et al.*, 2009; Pantin *et al.*, 2012). However, as changes in light and temperature can have direct effects on *E*, the data presented here suggests that a similar range of *E* should be maintained when testing for light and temperature effects on the hydraulic conductance of the extra-xylem pathway independent of variation in *E*. To date, the relative contribution of symplastic, transcellular, and apoplastic water transport through the extra-xylary tissues is still under debate (e.g. Tyree and Cheung, 1977; Tyree *et al.*, 1981; Johansson *et al.*, 1996; Morillon and Chrispeels, 2001; Aasamaa *et al.*, 2005; Aroca *et al.*, 2006; Cochard *et al.*, 2004; Cochard *et al.*, 2007; Kim and Steudle, 2007; Sellin *et al.*, 2008; Baaziz *et al.*, 2012; Rockwell *et al.*, 2014), as is the extent to which the different live tissues of a leaf (e.g. palisade and spongy mesophyll, bundle sheath, epidermis) participate in the transpiration stream (e.g. Zwieniecki *et al.*, 2007; Canny *et al.*, 2012).

Clearly one of the missing pieces of the puzzle is the location of the site(s) of evaporation inside the leaf (Pieruschka *et al.*, 2010; Peak and Mott, 2011). It is difficult to evaluate water transport through the extra-xylary pathways in the leaf if there is no clear understanding of where the liquid flow path ends and whether or not the locations of these evaporation sites vary with changes in the absolute rate of leaf water loss (i.e. *E*). For example, recent research suggests that vapour transport through the intercellular air spaces can account for a substantial amount of water transport between mesophyll cells and thus the hydraulic conductance of the extra-xylary component (Rockwell *et al.*, 2014; Buckley, 2014). Additionally, changes in *E* may shift the depth of the evaporation front within leaves (Rockwell *et al.*, 2014; Buckley, 2014) and alter the relative contribution of liquid and vapour transport through these parallel pathways within the mesophyll. Characterizing where the evaporation sites occur in the leaf is needed to fully understand how water transport through the mesophyll is partitioned between these parallels pathways (i.e. liquid–apoplastic, symplastic, transcellular; vapour–intercellular air spaces).

## Conclusions

The results presented here suggest that when plants are well-hydrated, *k*_leaf_ does not remain fixed or decrease as *E* increases, but rather increases with *E*. Here, this dynamic *k*_leaf_ is referred to as *k*_leaf(E)_, which incorporates the inherent *k*_leaf_ at zero *E*, *k*_leaf(0)_. This positive dependence of *k*_leaf_ on *E* tends to minimize or reduce water potential gradients along the soil–plant–atmosphere continuum. Minimizing variation in ΔΨ_stem–leaf_ over a broad range of *E* (i.e. maintaining isohydrodynamic conditions), and therefore maximizing leaf water content (LWC), has many potential implications for whole plant carbon balance. It is well recognized that decreases in Ψ_leaf_ and LWC can increase stomatal and biochemical limitations to CO_2_ assimilation rate (*A*) and thus decrease potential *A* for given environmental conditions (Lawlor, 2002; Lawlor and Cornic, 2002). A positive dependence of *k*_leaf_ on *E* will ultimately increase the range of stem water potentials where leaves can maintain water potential above the turgor loss point, supporting high LWC, *g*_s_, and *A*. This mechanism will dominate only while the xylem remains hydraulically intact, i.e. in well-hydrated leaves. As Ψ_leaf_ falls below the cavitation threshold the subsequent drop in *k*_xylem_ will dominate and the leaf will exhibit the classical pattern of falling *E* with declining *k*_leaf_. Minimizing ΔΨ_stem–leaf_ avoids other negative consequences of excessive water potential gradients such as reduced rates of export of photoassimilates from leaves (Nikinmaa *et al.*, 2013; Turgeon, 2010; Hölttä *et al.*, 2009; Hölttä *et al.* 2006; Thompson and Holbrook, 2004). The dynamic nature of *k*_leaf_ is therefore integral to many aspects of plant water use and productivity and should be considered in mechanistic vegetation models.

## Supplementary data

Supplementary data are available at *JXB* online.

Figure S1. Diurnal variation in photosynthetically active radiation (PAR, µmol m^–2^ s^–1^), stomatal conductance (*g*s, mol m^–2^ s^–1^), stem and leaf water potential (Ψstem and Ψleaf, MPa), leaf hydraulic conductance (*k*leaf, mmol m^–2^ s^–1^) and transpiration rate (*E*, mmol m^–2^ s^–1^) for three angiosperm species growing in a common garden.

Figure S2. Diurnal variation in photosynthetically active radiation (PAR, µmol m^–2^ s^–1^), stomatal conducatance (*g*s, mol m^–2^ s^–1^), stem and leaf water potential (Ψstem and Ψleaf, MPa), leaf hydraulic conductance (*k*leaf, mmol m^–2^ s^–1^), and transpiration rate (*E*, mmol m^–2^ s^–1^) for three gymnosperm species growing in a common garden.

Supplementary Data
