# Supplementary material for: Increasing leaf hydraulic conductance with transpiration rate minimizes the water potential drawdown from stem to leaf
Source: J Exp Bot. 2014 Dec 29;66(5):1303–15. doi: 10.1093/jxb/eru481 (PMC4339593; doi:10.1093/jxb/eru481)
Supplement: Supplementary Data [file supp_66_5_1303__index.html]

Increasing leaf hydraulic conductance with transpiration rate minimizes the water potential drawdown from stem to leaf — Increasing leaf hydraulic conductance with transpiration rate minimizes the water potential drawdown from stem to leaf — Supplementary Data 

# Increasing leaf hydraulic conductance with transpiration rate minimizes the water potential drawdown from stem to leaf

## Supplementary Data

Data files

**Files in this Data Supplement:**

- Supplementary Data - Supplementary Data
