# Supplementary material for: Increasing leaf hydraulic conductance with transpiration rate minimizes the water potential drawdown from stem to leaf
Source: J Exp Bot. 2014 Dec 29;66(5):1303–15. doi: 10.1093/jxb/eru481 (PMC4339593; doi:10.1093/jxb/eru481)
Supplement: Supplementary Data [file supp_eru481_jexbot131904_file001.pdf]

Increasing leaf hydraulic conductance with transpiration rate minimizes the water potential drawdown from stem to leaf.

Kevin A. Simonin, Emily Burns, Brendan Choat, Margaret M. Barbour, Todd E. Dawson and Peter J. Franks

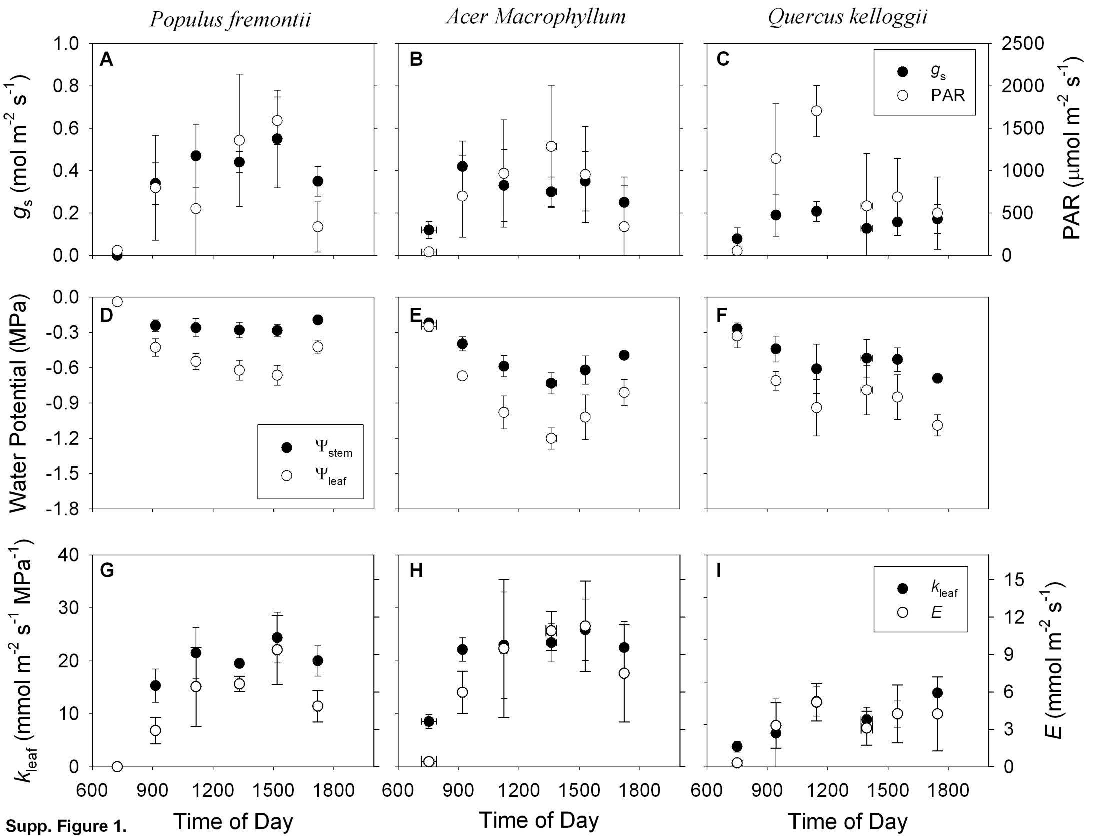

Supp. Figure 1.

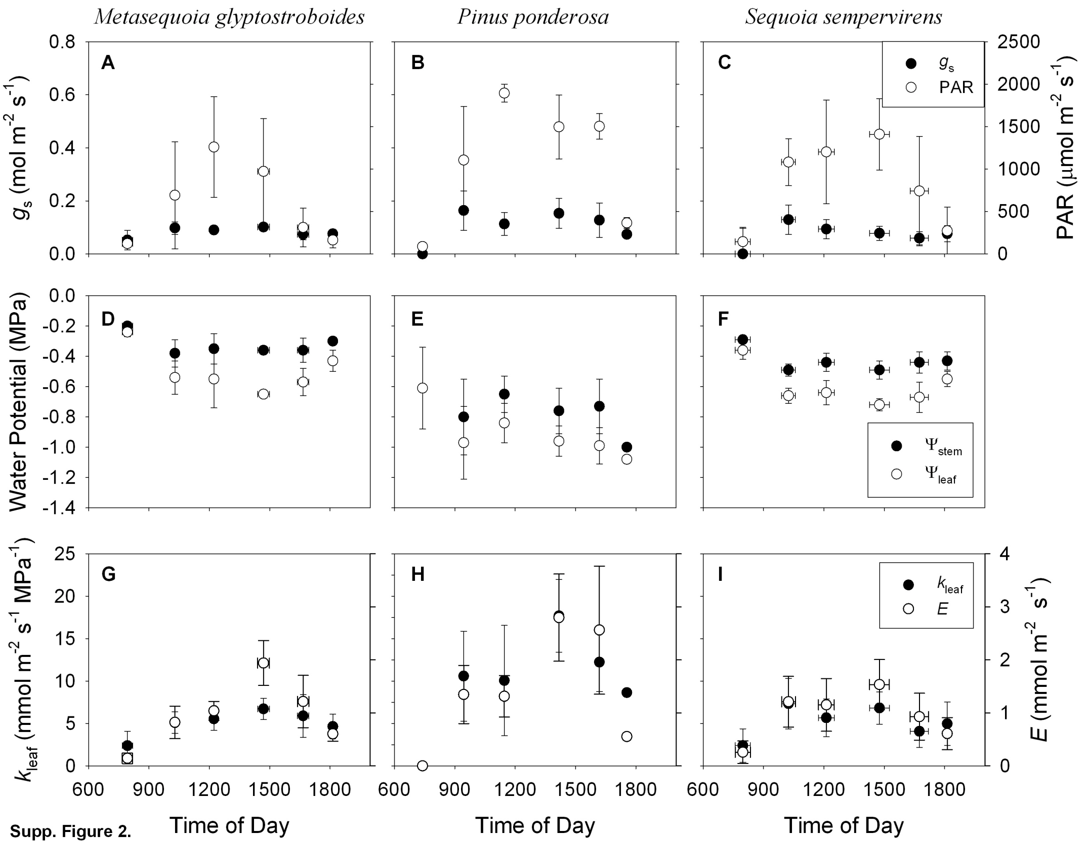

Supp. Figure 2.

Supplemental Figure 1. Diurnal variation in photosynthetically active radiation (PAR, umol m^{-2} s^{-1}) stomatal conducatance (g_s, mol m^{-2} s^{-1}), stem and leaf water potential (Ψ_{stem} and Ψ_{leaf}, MPa), leaf hydraulic conductance (k_{leaf}, mmol m^{-2} s^{-1}) and transpiration rate (E, mmol m^{-2} s^{-1}) for three angiosperm species growing in a common garden. Species are sorted by increasing leaf life span: (a, d, g) *Populus fremontii*, (b, e, h) *Acer macrophyllum,* and *Quercus kelloggii* (c, f, i).

Supplemental Figure 2. Diurnal variation in photosynthetically active radiation (PAR, umol m^{-2} s^{-1}) stomatal conducatance (g_s, mol m^{-2} s^{-1}), stem and leaf water potential (Ψ_{stem} and Ψ_{leaf}, MPa), leaf hydraulic conductance (k_{leaf}, mmol m^{-2} s^{-1}) and transpiration rate (E, mmol m^{-2} s^{-1}) for three gymnosperm species growing in a common garden. Species are sorted by increasing leaf life span: (a, d, g) *Metasequoia glyptostroboides,* (b, e, h) *Pinus ponderosa,* and (c, f, i) *Sequoia sempervirens*.
